# Evaluation of antibacterial efficacy of *Lawsonia inermis* Linn (henna) on periodontal pathogens using agar well diffusion and broth microdilution methods: An in-vitro study

**DOI:** 10.37796/2211-8039.1411

**Published:** 2023-09-01

**Authors:** Şevki Güler, Damla Torul, Sevda Kurt-Bayrakdar, Emine Kübra Tayyarcan, Çağrı Çamsarı, İsmail Hakkı Boyacı

**Affiliations:** a Private Practice, Güler Dent Samsun Oral and Dental Health Polyclinic, Samsun, Turkey; b Ordu University, Faculty of Dentistry, Department of Oral and Maxillofacial Surgery, Ordu, Turkey; c Eskişehir Osmangazi University, Faculty of Dentistry, Department of Periodontology, Eskişehir, Turkey; d Hacettepe University, Faculty of Engineering, Department of Food Engineering, Ankara, Turkey; e Bolu Abant İzzet Baysal University, Innovative Food Technologies Development Application and Research Center, Bolu, Turkey

**Keywords:** *A.actinomycetemcomitans*, Antibacterial, *Lawsonia inermis*, *P.gingivalis*, Plant

## Abstract

**Background:**

Although widely explored in medicine, limited evidence exists in the literature regarding the efficacy of *Lawsonia inermis* Linn (henna) in the dental field.

**Aim:**

This study aimed to investigate the antibacterial effect of henna on *Aggregatibacter actinomycetemcomitans* and *Porphyromonas gingivalis* in vitro.

**Methods:**

The agar well diffusion and broth microdilution methods were used to evaluate the antibacterial effect of henna extracts. Dimethyl sulfoxide was used to prepare the ethanol extract of henna, and distilled water was used to prepare the water extract. For both ethanol and water extracts, 4 different concentrations were prepared as 15, 30, 60, and 120 mg/mL.

**Results:**

It was determined that the water and ethanol extracts of the henna samples did not show an inhibition zone on *P.gingivalis* and *A.actinomycetemcomitans*. As a result of the evaluations made with the broth microdilution method, it was found that the ethanol extract had a higher inhibitory effect on both bacteria, and both extracts had more inhibitory effects against *A.actinomycetemcomitans*.

**Conclusion:**

To understand the effect of henna on periodontal pathogens, more comprehensive in vitro studies should be performed on henna samples at different concentrations and with different bases.

## 1. Introduction

The world has a rich plant heritage which is an important part of human society since the dawn of civilization [1]. When compared to the numerous side effects of synthetic medications in use, herbal medicine has gained attention due to its usage in traditional medicine, high safety margins, and cost-effectiveness [2,3]. Thus, there is an increasing trend in the use of medicinal plants in the field of medicine [4].

*Lawsonia inermis* Linn, also known as henna, is a 2-6-m-long flowering plant from the Lythraceae family. Henna, found in the tropics, is native to North Africa and Southeast Asia and is generally grown along the African coast of India, Iran, and the Mediterranean. For more than 9000 years, henna has been used in cosmetics and medicine [5–7]. Chemical components isolated from this plant include aliphatic derivatives, naphthoquinone derivatives, xanthones, phenolic compounds, terpenoids, coumarin, sterols, and fatty/amino acids [7,8]. Due to this rich chemical content, henna has been reported to be used therapeutically in conditions such as wounds, edema, headache, ulcer, and diseases such as diarrhea, jaundice, leprosy, bronchitis, menstrual disorder, rheumatism, dysentery, hemorrhoids, and skin problems [9].

To date, various studies have been conducted showing many pharmacological effects such as antimicrobial, analgesic, antipyretic, anti-inflammatory, antioxidant, antineoplastic, sedative, hepatoprotective, hypoglycemic, and immunomodulatory, of henna [3,7,10–17]. Although widely explored in medicine, limited evidence exists in the literature regarding the efficacy of henna in the dental field [2,3,18–22]. This study aims to explore the antibacterial effects of different henna extracts against periodontal pathogens *Aggregatibacter actinomycetemcomitans* and *Porphyromonas gingivalis*.

## 2. Materials & methods

### 2.1. Preparation of plant extracts

Two different extracts containing ethanol and water were used to determine the in vitro antibacterial activity of henna. The preparation protocol for the extracts is as follows;

- The L. *inermis* leaves (Xi’an XiaoCao Botanical Development Co., Ltd.) were mechanically pulverized into fine powder in an electric grinder. Powdered leaves (10 g) were added to two bottles of 100 mL volume and 50 mL of each solution was added to each bottle separately.
- The flasks were kept in the incubator at 180 rpm and 37 °C overnight.
- The contents of the bottle were first passed through a four-layer muslin cloth and then through Whitman filter paper.
- The filtered part was evaporated at 50 °C. The weight of the remains was recorded.
- Dimethyl sulfoxide was used to dissolve the residues of the ethanol extract, while the aqueous extract residues are dissolved in distilled water at different concentrations.
- The extracts were stored at 4 °C until used.

### 2.2. Bacteria and growth conditions

*A. actinomycetemcomitans* (ATCC® 33384 ™) and *P. gingivalis* (ATCC® 33277™) were used to examine the antimicrobial effect of henna extracts on oral pathogens. Brain Heart Infusion broth was used to propagate and prepare new stocks of *A. actinomycetemcomitans*. Bacteria were grown in an anaerobic jar in the presence of the Anaerocult® C (Merck Millipore Corporation, Darmstadt, Germany) at 37 °C for 24–48 h. ATCC® Medium 2722: Supplemented Tryptic Soy Broth was used for the propagation of *P. gingivalis*. Cultures were grown in an anaerobic jar in the presence of the Anaerocult ® A (Merck Millipore Corporation, Darmstadt, Germany) at 37 °C for 48–72 h. The stocks of the propagated bacteria were prepared with 50% glycerol and kept at −20 °C during the study.

### 2.3. Agar well diffusion method

The Agar well diffusion method was used to determine the antimicrobial effect of *L. inermis* extracts against *A. actinomycetemcomitans* and *P. gingivalis* [3,23,24]. For this purpose, the ethanol extract was dissolved using dimethyl sulfoxide and the water extract was dissolved using distilled water. Four different concentrations (15 mg/mL, 30 mg/ mL, 60 mg/mL, and 120 mg/mL) have been prepared for both ethanol and water extracts. Before the experiment, the bacteria were grown in Brain Heart Infusion and Supplemented with Tryptic Soy Broth for 18–24 h under the conditions specified in section 2.2 and dilutions were prepared with sterile physiological saline (0.85% NaCl) corresponding to the McFarland 0.5 standard (1–2 × 10^8^ CFU/mL). Then, 100 μL of the appropriate dilution was spread over the surface of Columbia Blood Agar (Sigma–Aldrich, St. Louis, MO) and wells were drilled on the agar using sterile cork borers. Twenty μL of different concentrations of henna extract were added to each well. As a negative control, the same amount of dimethyl sulfoxide for ethanol extracts and the same amount of sterile distilled water for water extracts were added to the wells. Plates were incubated in an anaerobic jar at 37 °C for 24–72 h. Following incubation, the inhibition zones around the wells were examined and the diameters of the zones was measured in millimeters. Each trial was repeated three times.

### 2.4. Determination of minimum inhibitory concentration (MIC) and minimum bactericidal concentration (MBC)

The broth microdilution method was applied to determine the minimum inhibitory concentration (MIC) and minimum bactericidal concentration (MBC) of *L. inermis* extracts against oral pathogens [25]. Briefly, two-fold dilutions of extracts ranging from 120 mg/mL to 0.47 mg/mL were prepared in a 96-well microtiter plate using a tryptic Soy Broth medium.

Then, 100 μL of cultures were added to these wells. The wells containing bacteria without any extracts were used as growth control. As a negative control, only the liquid medium and the *L. inermis* extracts were added to the wells. The microplates were incubated at the conditions given above and then examined for visible turbidity caused by bacterial growth. The lowest *L. inermis* concentration which inhibits visible bacterial growth was assessed as MIC. While determining the MBC values, appropriate dilutions of the samples taken from the wells were plated on agar to determine the number of bacteria, which remained alive after incubation. The lowest concentration at which 99.9% of the inoculum is killed was assessed as MBC. The experiments were repeated in triplicate for each pathogen.

## 3. Results

### 3.1. Antibacterial activity of henna extracts

**Fig. 1:**
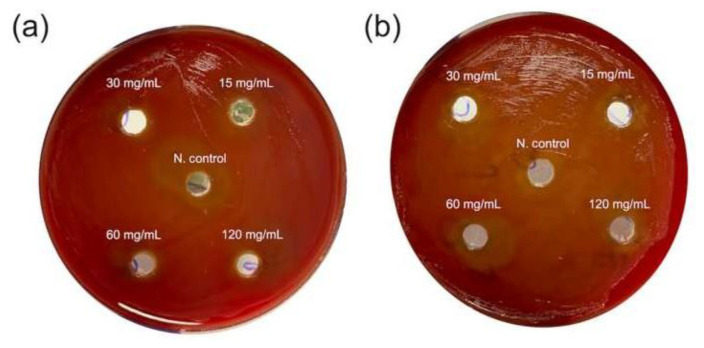
The effect of water (a) and ethanol (b) extracts of henna samples against *A. actinomycetemcomitans*.

As a result of experiments using the agar well diffusion method, petri dishes were examined and it was determined that water and ethanol extracts prepared at concentrations of 15, 30, 60, and 120 mg/mL of henna did not show an inhibition zone against *A. actinomycetemcomitans* (Fig. 1).

**Fig. 2:**
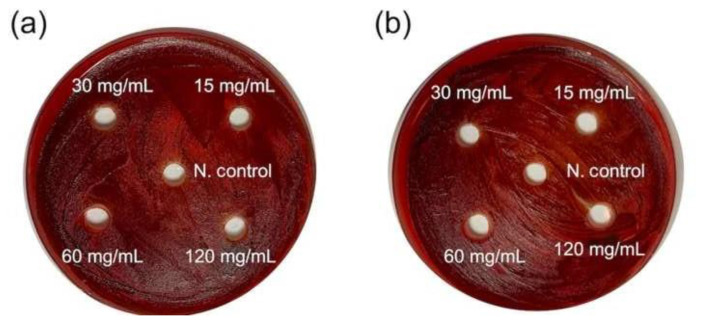
The effect of water (a) and ethanol (b) extracts of henna samples against *P. gingivalis*.

Similarly, it was found that water and ethanol extracts of henna prepared at concentrations of 15, 30, 60, and 120 mg/mL did not show an inhibition zone against *P. gingivalis* (Fig. 2).

As a result of the studies, it was determined that both water and ethanol extracts did not cause visible inhibition against both pathogens on agar (Table 1).

### 3.2. Determination of MIC and MBC

MICs and MBCs of the henna extracts were determined by using the broth microdilution method. As a result of the analysis, MICs of water and ethanol extract of henna against *Aggregatibacter actinomycetecomitans* were determined as 15 and 3.75 mg/mL, and MBCs as 60 and 15 mg/mL, respectively. Similarly, MICs of water and ethanol extract of henna against *P. gingivalis* were found to be 60 and 7.5 mg/mL, and MBCs as 120 and 15 mg/ mL, respectively (Table 2).

When the obtained results were examined, it was seen that the inhibition effect of the ethanol extract of henna was higher for both *A. actinomycetemcomitans* and *P. gingivalis* than that of water extract. Similarly, it was found that water and alcohol extracts of henna had a more inhibitory effect against *A. actinomycetemcomitans* than that of *P. gingivalis*.

## 4. Discussion

For thousands of years, plants have served as valuable components of the medicine, spices, beverages, cosmetics and, dyes to protect human health and to increase the quality of life. Research about herbal medicine has led to the discovery of the tremendous potential for herbal plants used in traditional systems [5,26]. Although many beneficial effects of henna on the medical field have been proven, the efficacy of henna in dentistry is not clear [3,7,9]. This study aimed to investigate the antibacterial effects of different henna extracts against periodontal pathogens *A. actinomycetemcomitans* and *P. gingivalis*.

When the studies in which antibacterial effectiveness evaluations are examined, it is seen that the effectiveness of different henna extracts on different bacteria in medicine is evaluated [17,24,27–32], but the number of studies evaluating the effect on periodontal pathogens is quite low [3]. Akter et al. evaluated the antibacterial activity of 3 different extracts of *L. inermis* (ethanol, petroleum ether, and chloroform) on 15 different bacteria (6 g-positive and 9 g-negative). In the results of this study, they reported that among the extracts tested, ethanol extract was found to have the highest antibacterial activity. Also, the diameter of the zone of inhibition showed that Gram-negative bacteria are more sensitive than Gram-positive bacteria to the extracts [27]. Although the well diffusion results were in line with the present study, the results of the previous study could not be directly compared with the present study due to the different strains of bacteria evaluated in the current study and the difference in the type and concentration of extract used. Habbal et al. evaluated the effect of fresh and dried leaves and seeds of henna obtained from Oman on *Staphylococcus aureus*, *Escherichia coli*, and *Pseudomonas aeruginosa*, they reported that henna had antibacterial activity on many different bacterial strains [17]. In another study, Hussain et al. evaluated the effect of aqueous methanolic *L. inermis* extracts on 2-g-negative and 3-g-positive bacteria and reported that the plant extracts had a great antibacterial potential against the evaluated bacteria [33].

Previously, the use of henna as an alternative natural agent in dentistry has come to mind, and a limited number of academic studies have been carried out for this purpose. It is seen that the use of henna as an antifungal agent in controlling infections such as candida has been investigated in the majority of studies [2,20,34]. Samadi et al., Sujanamulk et al. and Mardani et al. found promising results in their studies for this purpose [2,20,34].

Zubardiah et al. used oral solutions with *L. inermis* in patients with gingivitis in a study they carried out, and reported that this solution reduced the signs of gingivitis and that henna may be an alternative agent that can be used in the treatment of periodontal disease in the future [35]. In addition, they reported in this study that the therapeutic effect of these plant extracts may be due to their antibacterial activity on periodontal pathogens. However, in this study, the effect of applying henna solution at only 5 different concentrations on clinical indixes was examined and no antibacterial activity was evaluated [35].

When the literature is examined, it is seen that the most similar study with the current study is the Vahabi et al.’s study that evaluated the effectiveness of hydroalcoholic extracts of *L. inermis* on *A. Actinomycetemcomitans* by both agar well diffusion technique and broth microdilution technique [3]. Although only water and ethanol extracts were examined in the current study, Vahabi et al. similarly, observed that there was no antimicrobial activity of the Hydroalcoholic extract of *L. inermis*, in disc diffusion and agar well diffusion methods, but antimicrobial activity was observed in broth microdilution method [3]. In this study, the broth microdilution MIC of *L. inermis* was found to be 0.156 mg/ mL [3]. In our current study, MICs were found as 15 mg/mL and 3.75 mg/mL for water and ethanol extracts, respectively. It is thought that this may be due to the use of different *L. inermis* extracts and the difference in the preparation of plant extracts.

With more advanced techniques, the efficacy of different henna extracts on pathogens could be evaluated over a wider range of concentrations. This is a limitation of our study. Another limitation is the lack of more detailed studies to support its clinical use, such as cytotoxicity. As it is known, the primary etiological factor of periodontitis, which is a chronic inflammatory disease characterized by attachment loss and alveolar bone destruction, is microbial dental plaque [36–38]. There are a wide variety of periodontal pathogens involved in the pathogenesis of the disease, and some of the most well-known among these is the gram-negative bacteria *A. actinomycetemcomitans* and *P*. gingivalis [39]. As far as we know, there is only one study evaluating the antibacterial activity of the *L. inermis* on *A. actinomycetemcomitans*, while the study evaluating the effect on *P. gingivalis* has not been performed before. The present study is the first in the literature in this respect. However, to understand the effect of henna on periodontal and dental pathogens, more comprehensive in vitro studies should be performed on henna samples with different concentrations and different bases. In addition, evaluation of the possible effects of henna on wound healing of oral and periodontal tissues and its effects such as toxicity will provide more detailed information to the literature.

## Figures and Tables

**Table 1:** Inhibition effect of water and ethanol extracts of henna prepared in different concentrations against oral pathogens.

Bacteria	Extract concentration (mg/mL)	Inhibition zone (mm)	

		Water extract	Ethanol extract
*A. actinomycetemcomitans*	15	–	–
	30	–	–
	60	–	–
	120	–	–
*P. gingivalis*	15	–	–
	30	–	–
	60	–	–
	120	–	–

-; No inhibition observed.

**Table 2:** MICs and MBCs of henna extracts against *A. actinomycetecomitans* and *P. gingivalis*.

Bacteria	Water extract		Ethanol extract	
	
	MIC (mg/mL)	MBC (mg/mL)	MIC (mg/mL)	MBC (mg/mL)
*A. actinomycetemcomitans*	15.0	60.0	3.75	15.0
*P. gingivalis*	60.0	120.0	7.5	15.0

## References

[1] Elaguel A Kallel I Gargouri B Amor IB Hadrich B Mesaaoud EB Lawsonia inermis essential oil: extraction optimization by RSM, antioxidant activity, lipid peroxydation, and antiproliferative effects Lipids Health Dis 2019 18 1 196 31727081 10.1186/s12944-019-1141-1 PMC6857162

[2] Mardani M Badiee P Gharibnavaz M Jassebi A Jafarian H Ghassemi F Comparison of anti-Candida activities of the ancient plants Lawsonia inermis and Ziziphus spina christi with antifungal drugs in Candida species isolated from the oral cavity J Conserv Dent 2018 21 4 359 62 30122813 10.4103/JCD.JCD_291_17 PMC6080183

[3] Vahabi S Hakemi-Vala M Gholami S In vitro antibacterial effect of hydroalcoholic extract of Lawsonia inermis, malva sylvestris, and boswellia serrata on aggregatibacter actinomycetemcomitans Adv Biomed Res 2019 8 22 31016180 10.4103/abr.abr_205_18 PMC6446579

[4] Jafarzadeh L Seifi N Shahinfard N Sedighi M Kheiri S Shirzad H Antioxidant activity and teratogenicity evaluation of Lawsonia inermis in BALB/c mice J Clin Diagn Res 2015 9 5 1 4 10.7860/JCDR/2015/12290.5911 PMC4484084 26155492

[5] Makhija IK Dhananjaya D Kumar V Devkar R Khamar D Manglani N Lawsonia inermis-from traditional use to scientific assessment AJP Sci and Pharm 2011 2 1

[6] Sultana S Khosru KH Analgesic and antidiarrhoeal activities of Lawsonia inermis Int J Pharm Sci Res 2011 2 12 3183

[7] Ali BH Bashir AK Tanira MO Anti-inflammatory, antipyretic, and analgesic effects of Lawsonia inermis L. (henna) in rats Pharmacology 1995 51 6 356 63 8966192 10.1159/000139347

[8] Hekmatpou D Ahmadian F Eghbali M Farsaei S Henna (Lawsonia inermis) as an inexpensive method to prevent decubitus ulcers in critical care units: a randomized clinical trial J Evid Based Integr Med 2018 23 2515690X18772807. 10.1177/2515690X18772807 PMC5954567 29756474

[9] Gull I Sohail M Aslam MS Amin Athar M Phytochemical, toxicological and antimicrobial evaluation of Lawsonia inermis extracts against clinical isolates of pathogenic bacteria Ann Clin Microbiol Antimicrob 2013 12 36 24289297 10.1186/1476-0711-12-36 PMC4220702

[10] Mir NT Saleem U Anwar F Ahmad B Ullah I Hira S Lawsonia inermis markedly improves cognitive functions in animal models and modulate oxidative stress markers in the Brain Medicina (Kaunas) 2019 55 5 192 31121979 10.3390/medicina55050192 PMC6571555

[11] Inawati S Winarno H The effect of Inai (Lawsonia inermis Linn) leaves extract on blood sugar level: an experimental study Res J Pharmacol 2008 2 2 20 3

[12] Abdel-Hamid NM Mohafez OM Nazmy MH Farhan A Thabet K The effect of co-administration of Lawsonia inermis extract and octreotide on experimental hepatocellular carcinoma Environ Health Prev Med 2015 20 3 195 203 25726025 10.1007/s12199-015-0451-9 PMC4434234

[13] Mikhaeil BR Badria FA Maatooq GT Amer MM Antioxidant and immunomodulatory constituents of henna leaves Z Naturforsch C Biosci 2004 59 7–8 468 76 10.1515/znc-2004-7-803 15813363

[14] Zumrutdal E Karateke F Daglioglu K Gulkaya M Colak O Koksal F Lawsonia inermis-an alternative treatment for hyperthyroidism? Bratisl Lek Listy 2014 115 2 66 9 24601697 10.4149/bll_2014_014

[15] Nesa L Munira S Mollika S Islam M Evaluation of analgesic, anti-inflammatory and CNS depressant activities of methanolic extract of Lawsonia inermis barks in mice Avicenna J Phytomed 2014 4 287 25068143 PMC4110786

[16] Raja W Agrawal RC Ovais M Chemopreventive action of Lawsonia inermis leaf extract on DMBA-induced skin papilloma and B16F10 melanoma tumour Pharmacologyonline 2009 2 1243 9

[17] Habbal OA Al-Jabri AA El-Hag AH Al-Mahrooqi ZH Al-Hashmi NA In-vitro antimicrobial activity of Lawsonia inermis Linn (henna). A pilot study on the Omani henna Saudi Med J 2005 26 1 69 72 15756356

[18] Nawasrah A Gad MM El Zayat M Effect of henna addition on the surface roughness and hardness of poly-methylmethacrylate denture base material: an in vitro study J Contemp Dent Pract 2018 19 6 732 8 29959304

[19] Nawasrah A AlNimr A Ali AA Antifungal effect of henna against Candida albicans adhered to acrylic resin as a possible method for prevention of denture stomatitis Int J Environ Res Publ Health 2016 13 5 10.3390/ijerph13050520 PMC4881145 27223294

[20] Sujanamulk B Chintamaneni R Chennupati A Nahar P Chaluvadi RS Vemugunta R Evaluation of antifungal efficacy of ethanolic crude lawsone and listerine mouthwash in uncontrolled diabetics and denture wearers - a randomized clinical trial J Clin Diagn Res 2016 10 6 90 5 10.7860/JCDR/2016/19463.8036 PMC4963779 27504419

[21] Bhavana S Nahar P Lakshmi CR Kilaru NB Vemugunta R A randomized clinical trial to assess and compare the antimicrobial activity of plants of Lythraceae family with hiora mouth washes in subjects with chronic Periodontitis– “Unveiling the unseen effects” Int J Pharm Sci 2017 8 5 2184 93

[22] Lies Z Janti S Effectiveness of Lawsonia inermis L. Leaves methanol extracts on gingivitis healing (in vivo study on sprague dawley rats) BJMMR 2016 1 8

[23] Bali EB Açõk L Akca G Sarper M Elçi PM Avcu F Antimicrobial activity against periodontopathogenic bacteria, antioxidant and cytotoxic effects of various extracts from endemic Thermopsis turcica Asian Pac J Trop Biomed 2014 4 7 505 14 25183268 10.12980/APJTB.4.2014APJTB-2013-0010 PMC4032822

[24] Jeyaseelan EC Jenothiny S Pathmanathan M Jeyadevan J Antibacterial activity of sequentially extracted organic solvent extracts of fruits, flowers and leaves of Lawsonia inermis L. from Jaffna Asian Pac J Trop Biomed 2012 2 10 798 802 23569850 10.1016/S2221-1691(12)60232-9 PMC3609233

[25] Saquib SA AlQahtani NA Ahmad I Kader MA Al Shahrani SS Asiri EA Evaluation and comparison of antibacterial efficacy of herbal extracts in combination with antibiotics on periodontal pathobionts: an in vitro microbiological study Antibiotics 2019 8 3 89 31266146 10.3390/antibiotics8030089 PMC6783985

[26] Berenji F Rakhshandeh H Ebrahimipour H In vitro study of the effects of henna extracts (Lawsonia inermis) on Malassezia species Jundishapur J Microbiol 2010 3 3 125 8

[27] Akter A Neela F Khan M Islam M Alam M Screening of ethanol, petroleum ether and chloroform extracts of medicinal plants, Lawsonia inermis L. and Mimosa pudica L. for antibacterial activity Indian J Pharmaceut Sci 2010 72 3 388 10.4103/0250-474X.70492 PMC3003179 21188055

[28] Bhuvaneswari K Gnana PS Kuruvilla A Appala RB Inhibitory concentrations of Lawsonia innermis dry powder for urinary pathogens Indian J Pharmacol 2002 34 4 260

[29] Habbal O Hasson S El-Hag A Al-Mahrooqi Z Al-Hashmi N Al-Bimani Z Antibacterial activity of Lawsonia inermis Linn (henna) against Pseudomonas aeruginosa Asian Pac J Trop Biomed 2011 1 3 173 6 23569753 10.1016/S2221-1691(11)60021-X PMC3609186

[30] Nauman K Arshad M Screening of aqueous methanol plant extracts for their antibacterial activity Science and Technology against Microbial Pathogens: Research, Development and Evaluation World Scientific 2011 123 7

[31] Nigussie D Davey G Legesse BA Fekadu A Makonnen E Antibacterial activity of methanol extracts of the leaves of three medicinal plants against selected bacteria isolated from wounds of lymphoedema patients BMC Complement Med Ther 2021 21 1 1 10 33390165 10.1186/s12906-020-03183-0 PMC7778819

[32] Muhammad H Muhammad S The use of Lawsonia inermis Linn.(henna) in the management of burn wound infections Afr J Biotechnol 2005 4 9

[33] Hussain T Arshad M Khan S Sattar H Qureshi M In vitro screening of methanol plant extracts for their antibacterial activity Pakistan J Bot 2011 43 531 8

[34] Samadi FM Suhail S Sonam M Sharma N Singh S Gupta S Antifungal efficacy of herbs J Oral Biol Craniofac Res 2019 9 1 28 32 30197861 10.1016/j.jobcr.2018.06.002 PMC6126409

[35] Zubardiah L Mustaqimah DN Auerkari EI Effectiveness of Lawsonia inermis linneaus leaves infusion in gingivitis healing Dentika Dental J 2012 17 2 105 10

[36] Offenbacher S Periodontal diseases: pathogenesis Ann Periodontol 1996 1 1 821 78 9118282 10.1902/annals.1996.1.1.821

[37] Flemmig TF Periodontitis Ann Periodontol 1999 4 1 32 7 10863373 10.1902/annals.1999.4.1.32

[38] Sánchez AR Kupp LI Sheridan PJ Sánchez DR Maternal chronic infection as a risk factor in preterm low birth weight infants: the link with periodontal infection J Int Acad Periodontol 2004 6 3 89 94 15368875

[39] Mombelli A Casagni F Madianos PN Can presence or absence of periodontal pathogens distinguish between subjects with chronic and aggressive periodontitis? A systematic review J Clin Periodontol 2002 29 10 21 12787203 10.1034/j.1600-051x.29.s3.1.x

